# Exploring the Diversity of Visceral, Subcutaneous and Perivascular Adipose Tissue in a Vascular Surgery Population

**DOI:** 10.3390/jcdd10070271

**Published:** 2023-06-25

**Authors:** Joana Ferreira, Julieta Afonso, Alexandre Lima Carneiro, Isabel Vila, Cristina Cunha, Susana Roque, Cristina Silva, Amílcar Mesquita, Jorge Cotter, Margarida Correia-Neves, Armando Mansilha, Adhemar Longatto-Filho, Pedro Cunha

**Affiliations:** 1 Vascular Surgery Department, Centro Hospitalar de Trás-os-Montes e Alto Douro, 5000-508 Vila Real, Portugal; 2 Life and Health Science Research Institute (ICVS), School of Medicine, University of Minho, 4710-057 Braga, Portugal; 3 Academic Center Hospital da Senhora da Oliveira, 4835-044 Guimarães, Portugal; 4 ICVS/3B’s—PT Government Associated Laboratory, 4710-057 Braga, Portugal; 5 Clinical Academic Center Hospital de Trás-os-Montes e Alto Douro, Professor Doutor Nuno Grande, CAC^TMAD^, 5000-508 Vila Real, Portugal; 6 Radiology Department, Unidade Local de Saúde Alto Minho, 4904-858 Viana do Castelo, Portugal; 7 Medicine Department, Hospital da Senhora da Oliveira, 4835-044 Guimarães, Portugal; 8 Center for the Research and Treatment of Arterial Hypertension and Cardiovascular Risk, Internal Medicine Department, Hospital da Senhora da Oliveira, 4835-044 Guimarães, Portugal; 9 Vascular Surgery Department, Hospital da Senhora da Oliveira, 4835-044 Guimarães, Portugal; 10 Faculty of Medicine, University of Porto, 4200-319 Porto, Portugal; 11 Department of Angiology and Vascular Surgery, Hospital de São João, 4200-319 Porto, Portugal; 12 Faculty of Medicine, Department of Pathology, University of São Paulo, São Paulo 05508-900, SP, Brazil; 13 Molecular Oncology Research Center, Barretos Cancer Hospital, Barretos 14784-390, SP, Brazil

**Keywords:** adipose tissue, inflammation, cardiovascular risk factors, statins, angiotensin-converting-enzyme inhibitors, angiotensin receptor antagonist

## Abstract

The prevalence of obesity has doubled, with a concomitant increase in cardiovascular disease. This study aimed to compare the characteristics of visceral, subcutaneous and peri-aortic adipose tissue determined with computed tomography (CT) scans and to correlate them with cardiovascular risk factors, anthropometric measures and medication. An observational and prospective study was conducted, and 177 subjects were included. Peri-aortic adipose tissue had the highest density, while the subcutaneous adipose tissue had the lowest. The density of subcutaneous adipose tissue differs from the density of visceral (*p* = 0.00) and peri-aortic adipose tissue (*p* = 0.00). Smokers/ex-smokers had a lower area (*p* = 0.00) and density (*p* = 0.02) of subcutaneous adipose tissue. Multiple linear regression analysis showed that sex was a predictor of subcutaneous adipose tissue area (β = −0.27, t = −3.12, *p* = 0.00) but smoking habits were not. After controlling for sex, we found that the association between smokers/ex-smokers and area of subcutaneous adipose tissue was lost, but the association with density persisted. Patients with hypertension had a higher visceral adipose tissue area, and this relationship was maintained even after adjusting for gender. Peri-aortic adipose tissue is similar to visceral and distinct from subcutaneous adipose tissue. Cardiovascular risk factors have different influences in distinct adipose compartments.

## 1. Introduction

Over the past several decades, the prevalence of obesity has doubled, with a concomitant increase in cardiovascular disease [1,2]. In 2030, almost 20% of the population will be obese [3]. Obesity contributes directly to incident cardiovascular risk factors [2]. Obesity also leads to the development of cardiovascular disease and cardiovascular mortality independently of other cardiovascular risk factors [2]. Longitudinal studies have demonstrated that the association between obesity and cardiovascular disease is not fully explained by traditional risk factors, as some obese patients are metabolically healthy [1]. This underscores the complex biology of adipose tissue and suggests that other factors, such as inflammation and unidentified factors, may also play a role in the disease process [1]. For instance, it has been reported that severely obese patients have a preservation of vascular function, due to the greater mobilization of endothelial progenitor cells [4,5].

On the other hand, more important than the total amount of adipose tissue is the regional fat distribution [6,7,8]. Two large compartments are classically identified: visceral adipose tissue and subcutaneous adipose tissue [9]. They can be estimated from anthropometric measures and rigorously quantified with computerized tomography (CT) scans. The two tissues have differences in structure (i.e., vascular density and innervation), adipokine expression profiles and thus metabolic functions [6,10,11]. Visceral adipose tissue surrounds the intra-abdominal organs [12,13]. Abdominal visceral fat has higher metabolic and secretion activity, with a more acute inflammatory profile compared to peripheral fat tissue [9,11]. Excessive visceral fat deposition is highly associated with dyslipidemia, diabetes mellitus (DM) and hypertension, resulting in atherosclerotic cardiovascular disease [14]. This explains why predominantly visceral fat accumulation is considered a more deleterious adipose tissue depot and a more powerful predictor of endothelial dysfunction than subcutaneous adiposity (9). Subcutaneous adipose tissue lies beneath the skin [12]. It has a protective role against cardiometabolic disease [11]. Subcutaneous adipose tissue depots show an enhanced adipocyte generation capacity compared with visceral adipose tissue [11]. This may be a protective property against metabolic dysfunction [11]. However, obesity does not only affect the visceral adipose tissue depots but also disturbs the subcutaneous adipose tissue equilibrium [11]. Perivascular adipose tissue (PVAT) is a particular fat depot that surrounds almost all blood vessels, including the abdominal aorta (peri-aortic adipose tissue) [11,15,16]. Adipocytes of the PVAT are not separated from the blood vessel wall by a fascial layer but encroach into the adventitial region [1,13,15]. PVAT is also connected to the vessel wall through microvessels [1,15,17]. Consequently, PVAT is a key adipose tissue depot in the context of cardiovascular disease and obesity, due to its intimate contact with the arterial wall [11]. 

Since different adipose tissue compartments have distinct biological characteristics and effects on the disease pathologies, the primary objective of this study was to compare the characteristics of the different adipose tissue depots (visceral, subcutaneous and peri-aortic) determined with CT scanning. The second purpose was to correlate the characteristics of adipose tissue with cardiovascular risk factors, anthropometric measurements and medication.

## 2. Materials and Methods

### 2.1. General Description

An observational and prospective study was conducted from January 2018 to December 2021 at the Vascular Surgery and Internal Medicine Departments—Hospital da Senhora da Oliveira Guimarães. The recruitment of patients was limited in 2020 due to the lockdowns and the decrease in hospital activities caused by the COVID-19 pandemic. 

The study population included patients with peripheral arterial disease, patients with varicose veins and patients with carotid stenosis, consecutively observed by vascular surgeons at our hospital consultations or at admission in the emergency room.

The variables collected were comorbidities, anthropometric measurements and histology of adipose tissue in patients with indication for surgery. A CT scan was performed at admission to rigorously quantify the amount of adipose tissue. 

### 2.2. Ethical Considerations

Ethics approval for data collection and cohort evaluation were obtained from the Ethics Committee of the Hospital Senhora da Oliveira—Guimarães (75/2017). The study was conducted according to Helsinki declaration, national and European guidelines for clinical research. The confidentiality of clinical records has been assured for both patients’ information and processing of biological samples. All the participants signed the informed consent. For each participant, a coded record form was creating detailing the data collected, to ensure blinding. 

### 2.3. Inclusion and Exclusion Criteria

Patients with vascular disease (peripheral arterial disease, varicose veins and carotid stenosis) were included in this study. Those with peripheral arterial disease were admitted if the clinician found that the individuals had symptoms of peripheral arterial disease confirmed with ankle brachial index. Patients with varicose veins were included based on the clinical judgement, while subjects with carotid stenosis were admitted if their carotid ultrasound identified an hemodynamic stenosis higher than 50%. The exclusion criteria were as follows: bedridden status or refusing to participate in the protocol; diseases responsible for body composition changes or pro-inflammatory state; recent diet changes; active malignancy; auto-immune disease; active infection; chronic renal failure in stage 4 (glomerular filtration rate—GFR < 30 mL/min/1.73 m^2^); history of coronary heart disease; and history of heart failure in the past three months. The glomerular filtration rate was calculated using the Cockcroft–Gault formula: GFR (mL/min/1.73 m^2^) = (140 − age) × (weight) × (0.85 if female)/(72 × serum creatinine) [18].

### 2.4. Clinical Characteristics

The clinical data collected were on patient’s age and sex, arterial hypertension, DM, dyslipidemia, smoking habits, coronary artery disease and cerebrovascular disease. Arterial hypertension was defined as requiring treatment with oral antihypertensive agents or as systolic blood pressure ≥140 mmHg and/or diastolic ≥90 mmHg [19]. DM was defined as the use of anti-diabetic medications, fasting glycaemia ≥126 mg/dL or glycated hemoglobin (HbA1c) ≥6.5% [20]. The diagnosis of dyslipidemia was described as requiring treatment with statins, an LDL cholesterol ≥70 mg/dL or plasma triglycerides ≥150 mg/dL [21]. Smoking habits were classified as active smoker, former smoker or non-smoker and quantified in pack-years (PY) (multiplying the number of packs of cigarettes smoked per day by the number of years the person has smoked). Former smoker status was defined as smoking cessation for at least six months [22]. Coronary artery disease was defined as angina, history of myocardial infarction, percutaneous coronary interventions and coronary artery bypass graft surgery [23]. Medication taken was also recorded.

### 2.5. Anthropometric Measurements

The anthropometric measurements were collected in the morning, after overnight fasting according to the procedure standardization [24]. They were measured after 5 min of rest in a comfortable room, with patients in light clothing, by a trained nurse blinded to the participants’ characteristics. 

The height was measured using a stadiometer (Seca 703 S, manufactured by seca in Hamburg, Germany). Participant stood on the stadiometer, facing forwards, as tall and straight as possible with their arms hanging loosely at their sides [24]. Each participant’s head was in the “Frankfort plane” (an imaginary line from the center of the ear hole to the lower border of the eye socket) [24]. The measurement was read to the nearest 1 mm when participants took a deep breath and held. The average of 3 measures was used [24].

The weight was determined using Seca 703S digital physician’s scale to the nearest 0.1 kg. 

Body mass index (BMI) was calculated by dividing the weight by height squared (kg/m^2^) [25].

Waist circumference (WC) was measured at the approximate midpoint between the lower margin of the last palpable rib and the top of the iliac crest in the horizontal position, using an anthropometric tape [26]. The subject was standing with arms at their sides, feet positioned close together and weight evenly distributed across the feet. The measurements were taken at the end of a normal expiration, when the lungs are at their functional residual capacity. Readings were rounded up to the nearest 0.1 cm [25].

The hip circumference measurement was taken around the widest portion of the buttocks [26]. The participants stood with arms at their sides, feet positioned close together and weight evenly distributed across the feet. Readings were rounded up to the nearest 0.1 cm [25].

Waist-to-hip ratio (WHR) was calculated as WC divided by hip circumference. 

Skinfold thickness was measured using a Harpenden Skinfold Caliper (manufactured by Bowers Group in West Sussex, United Kingdom). The measurements were taken on healthy, undamaged and uninfected dry skin on the right side. The skinfold was firmly grasped by the thumb and index finger, using the pads at the tip of the thumb and finger. The skinfold was pulled away from the body. The caliper was placed perpendicular to the fold, on the site marked, at approximately 1 cm below the finger and thumb, while maintaining the grasp of the skinfold, allowing the caliper to be released so that the full tension was placed on the skinfold. The dial was read to the nearest 0.5 mm, 1 to 2 s after the grip had been fully released. Two measurements were taken at each site. The final value recorded was the average of the two. 

The skinfolds for which thickness was determined were as follows [9]:

- Biceps—the anterior surface of biceps midway between the anterior fold and the antecubital fossa;
- Triceps—a vertical fold on the posterior midline of the upper arm, over the triceps muscle, halfway between the acromion process and olecranon process;
- Subscapular—taken on the diagonal line coming from the vertebral border to between 1 and 2 cm from the inferior angle of scapulae;
- Suprailiac—a diagonal fold above the crest of the ilium at the spot where an imaginary line would come down from the anterior auxiliary line just above the hipbone and 2–3 cm forward.

### 2.6. Determination of Adipose Tissue with CT Scan

A transverse CT image was obtained at the lower border of the third lumbar vertebra to quantify the visceral, subcutaneous and peri-aortic adipose tissue [27]. 

The area of subcutaneous compartment was calculated as the area of tissue between the visceral cavity and the body contour [28]. Visceral fat area was the area of the tissue that contours the visceral cavity [28].

**Figure 1:**
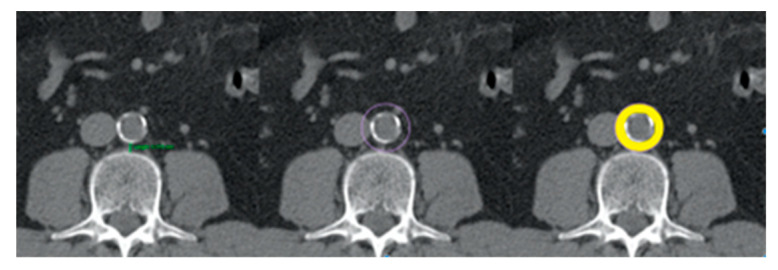
Abdominal CT scan showing the peri-aortic adipose tissue identified by the yellow circle. The method used to calculate the peri-aortic adipose tissue is schematically presented.

Peri-aortic fat area was determined by a concentric circle around the aorta [29]. The distance between the posterior border of the abdominal aorta and the 3rd lumbar vertebra (AVD—aorta vertebra distance) is a surrogate for the amount of PVAT. The PVAT has a uniform thickness around the vessel. The radius of the circle will thus be equal to the sum of the radius of the aorta and the AVD. PVAT area was calculated by subtracting the aortic area from the area of the circle (Figure 1), minus any intervening structures (e.g., lymph modes, small vessels).

Images were obtained for all subjects using a 128-slice CT scanner (Siemens Somatom Perspective 128, manufactured by Siemens Healthcare, Unipessoal, Lda in Lisbon, Portugal) after calibration with a phantom (CATPHAN^®^ water phantom, manufactured by Phantom Laboratory in New York, United States of America). CT scan was performed at the level L3-L4. Routine calibration was performed before quantitative computed tomography measurement. The scan parameters were as follows: 130 kV, 200 mAs, 45 cm FOV, 190 bed height, 5 mm slice thickness, 512 × 512 matrix, 64 × 0.6 mm detector collimation and 0.6 pitch. All subjects were scanned in the supine position with both arms stretched above the head in inspiratory apnea. The positional laser was at the intermammillary line. 

A radiologist and a vascular surgeon blinded to the subjects’ characteristics identified the L3 landmark, extracted the corresponding single cross-sectional image contained within a CT study and copied the image to a storage device (CDROM). The adipose tissue area and density (mass) were semi-automatically determined using Fiji (Fiji is just ImageJ), an open-source image-processing package based on ImageJ 1.52p^®^ [30]. The contours of the different adipose tissue compartments were traced using the semi-automated functions of the software. The Hounsfield units (HU) were measured from the density of the adipose tissue on CT images [30].

### 2.7. Samples of Subcutaneous Adipose Tissue

At the time of surgery, samples of subcutaneous adipose tissue were collected from the neck (in patients with indication to carotid endarterectomy) and from femoral region (patients submitted to femoral artery intervention or patients with indication for sapheno-femoral ligation). These samples were submitted to a protocol of immunohistology analysis with the antibodies CD 163+ and CD 45+ to identify macrophages and leucocyte, respectively (described in Appendix A). 

### 2.8. Analytic Evaluation

Peripheral blood was collected in the morning, after fasting overnight. It was processed in the Clinical Pathology Department of the Hospital Senhora da Oliveira, Guimarães. The following analysis were performed: glycosylated hemoglobin A1c (HbA1c), total cholesterol, high-density lipoprotein (HDL) cholesterol, triglycerides and low-density lipoprotein (LDL) cholesterol, using enzymatic assays.

### 2.9. Statistical Analysis

Continuous variables were expressed as the mean ± standard deviation and as the percentage for categorical variables. The Shapiro–Wilk test was used to assess all continuous variables for normality, and the homogeneity of variance was tested using Levene’s test. Continuous variables between two groups were compared with Student’s *t*-test or with Mann–Whitney. Continuous, non-normal variables between three groups were compared with Kruskal–Wallis. E2 measured the effect size, based on the statistic test for Kruskal–Wallis—H value. The post hoc comparison of the characteristics of visceral, subcutaneous and peri-aortic adipose tissue depots was performed using the Bonferroni test. Categorical variables between two groups were compared with Chi-square. Spearman’s correlation was utilized for measuring the strength and the relationship between two non-parametric measures. An analysis of Quade’s ANCOVA was used to adjust for sex the differences in the characteristics of the subcutaneous, visceral and peri-aortic adipose tissue compartments according to the presence of cardiovascular risk factors.

A *p*-value of less than 0.05 was considered significant. Statistical evaluation was performed using the Statistical Package for the Social Sciences (SPSS) software, version 20.0 (SPSS, Inc., Chicago, IL, USA). 

Sample size: To assess the differences in area and density among subcutaneous, visceral and peri-aortic adipose tissue, the main objective of this paper, a minimum of 158 patients is needed to identify an effect size of 0.25 with a power of 0.80.

## 3. Results

### 3.1. General Description of the Studied Population

This research work included 177 subjects (mean age: 67.2 ± 9.9 years old; 80.2% males), comprising 119 patients with peripheral arterial disease, 11 patients with carotid stenosis higher than 50% and 47 patients with varicose veins. The most prevalent cardiovascular risk factors were hypertension, smoking habits and dyslipidemia (Table 1). 

### 3.2. Characteristics of Visceral, Subcutaneous and Peri-Aortic Adipose Tissue Depots in CT Scan

**Figure 2:**
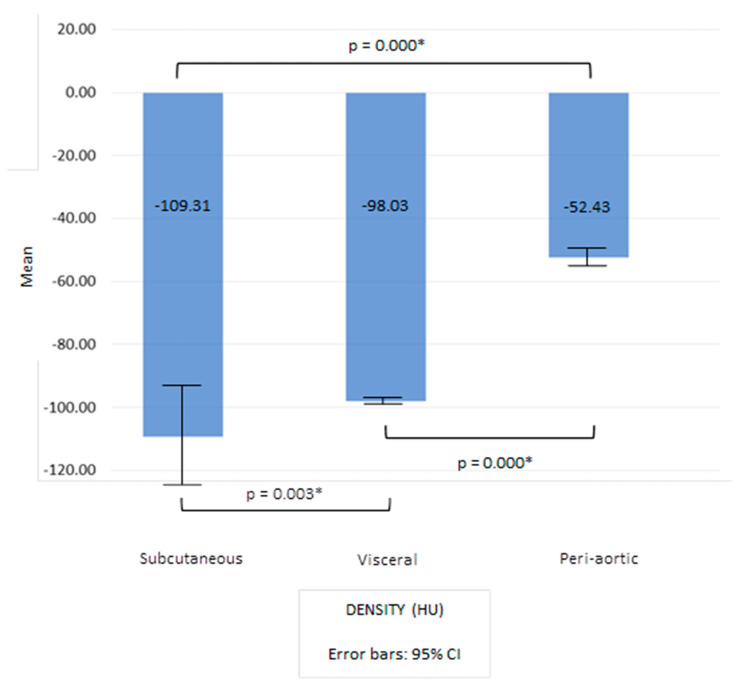
Density of subcutaneous, visceral, and peri-aortic adipose tissue in the studied population. Application of Kruskal–Wallis test. Density of subcutaneous and perivascular adipose tissue does not have a normal distribution, as determined with Shapiro–Wilk test. * *p* < 0.05.

In this studied population, subcutaneous adipose tissue had the lowest density, while the peri-aortic had the highest density (Figure 2). The Kruskal–Wallis test showed that there was a statistically significant difference between the densities of peri-aortic, visceral and subcutaneous adipose tissue (H(2) = 257.3, *p* = 0.00) with a strong effect size (Ε2 = 0.6) (Figure 2). The post hoc comparisons using the Bonferroni test demonstrated that the density of subcutaneous adipose tissue differs from the density of visceral tissue (*p* = 0.00) and from the density of the peri-aortic adipose tissue (*p* = 0.00). No difference was found between the densities of the visceral and the peri-aortic adipose tissue (*p* = 0.61).

The density of the visceral adipose tissue was moderately correlated with the density of the subcutaneous tissue and with the density of the peri-aortic adipose tissue (ρ = 0.47, *p* = 0.00; ρ = 0.50, *p* = 0.00, respectively). The density of the subcutaneous adipose tissue was very weakly correlated with the density of the peri-aortic adipose tissue (ρ = 0.19, *p* = 0.042).

The area of visceral adipose tissue was weakly correlated with the area of the subcutaneous tissue and with the area of the peri-aortic adipose tissue (ρ = 0.34, *p* = 0.00; ρ = 0.35, *p* = 0.00, respectively). The area of subcutaneous adipose tissue was also weakly correlated with the area of the peri-aortic adipose tissue (ρ = 0.26, *p* = 0.00).

### 3.3. Characteristics of Adipose Tissue Depot and Cardiovascular Risk Factors

The most prevalent cardiovascular risk factors were hypertension, smoking habits and dyslipidemia (Table 1). 

#### 3.3.1. Subcutaneous Adipose Tissue

We analyzed the association between the characteristics of subcutaneous adipose tissue and the cardiovascular risk factors. We noted that smokers/ex-smokers had a lower area and density of subcutaneous adipose tissue (Table 2 and Table 3). Smoking load was weak and negatively correlated with the area of subcutaneous adipose tissue (ρ = −0.25; *p* = 0.01). Patients with DM had a higher subcutaneous adipose tissue density (Table 3). No correlation was found between the characteristics of subcutaneous adipose tissue and HbA1c nor between subcutaneous adipose tissue area and lipid profile.

A positive and very weak correlation was found between subcutaneous adipose tissue area and the number of cardiovascular risk factors (ρ = 0.19; *p* = 0.02). No correlation was found between the density of subcutaneous adipose tissue and the number of cardiovascular risk factors. 

The multiple linear regression assessed the ability of smoker/ex-smoker status, sex, age, statins and antiplatelet therapy to predict the area of subcutaneous adipose tissue. The results reveal that sex was a significant predictor of subcutaneous adipose tissue area (β = −0.27, t = −3.12, *p* = 0.00). Antiplatelet therapy was also a significant predictor of subcutaneous adipose tissue area (β = −0.17, t = −2.07, *p* = 0.04). Smoker/ex-smoker status, age and statins had no impact on subcutaneous adipose tissue area (β = −0.187, t = −1.91, *p* = 0.06; β = −0.09, t = −1.06, *p* = 0.29; and β = 0.08, t = −0.96, *p* = 0.34, respectively). 

The multiple linear regression was also used to analyze the influence of smoker/ex-smoker status, DM, sex, age, statins and antiplatelet therapy on the density of subcutaneous adipose tissue. The results reveal that DM was a significant predictor of subcutaneous adipose tissue density (β = 0.278, t = 3.138, *p* = 0.002). The other variables had no impact. 

We also decided to adjust our data for sex. We found that smokers/ex-smokers have lower density of subcutaneous adipose tissue, after controlling for sex [F(1,134) = 12.88; *p* = 0.00]. However, the association between smokers/ex-smokers and subcutaneous adipose tissue area was lost after adjusting for gender [F(1,134) = 2.28; *p* = 0.13]. Patients with DM had a higher subcutaneous adipose tissue density, and the association persisted after adjusting for sex [F(1,137) = 16.19; *p* = 0.00].

For 68 patients with surgical indication, samples of subcutaneous adipose tissue were collected and submitted to an immunohistochemical analysis. We noted the following:

(a) Smokers/ex-smokers had a higher number of CD 163+ macrophages on the subcutaneous adipose tissue (CD 163+ macrophages more than moderate in 22.8% of smokers/ex-smokers and 11.2% of non-smokers, *p* = 0.04).
(b) Older patients had a higher number of CD 45+ leucocytes in the subcutaneous adipose tissue (CD 45+ leucocytes mild or absent: MD = 68.0 years old, AI = 13; CD 45+ leucocytes higher than moderate: MD = 70.0 years old, AI = 13, *p* = 0.03).

No other differences were found.

#### 3.3.2. Visceral Adipose Tissue

Analyzing the association between visceral adipose tissue and cardiovascular risk factors, we registered that patients with hypertension had a larger area of visceral adipose tissue (Table 4). No difference was found in visceral adipose tissue density and cardiovascular risk factor.

No correlation was found between the visceral adipose tissue area or density and smoking load or HbA1c. Serum triglycerides were weakly and positively correlated with visceral adipose tissue area (ρ = 0.26; *p* = 0.00). Serum triglycerides were weakly and negatively correlated with visceral adipose tissue density (ρ = −0.25; *p* = 0.00). No correlation was found between total cholesterol, LDL cholesterol, HDL cholesterol and visceral adipose tissue area or density.

A positive and weak correlation was found between visceral adipose tissue area and the number of cardiovascular risk factors (ρ = 0.34; *p* = 0.00). A negative and weak correlation was found between visceral adipose tissue density and the number of cardiovascular risk factors (ρ = −0.27; *p* = 0.00).

The multiple linear regression assessed the ability of hypertension, sex, age, triglycerides, statins and antiplatelet therapy to predict the area of visceral adipose tissue. The results reveal that sex, hypertension and triglycerides were significant predictors of visceral adipose tissue area (β = 0.27, t = 3.23, *p* = 0.00; β = 0.21, t = 2.46, *p* = 0.02; and β = 0.21, t = 2.54, *p* = 0.012, respectively). The other variables had no impact. 

After adjusting for gender, we found that patients with hypertension had a higher visceral adipose tissue area when compared to patients without hypertension [F(1,138) = 6.41; *p* = 0.01]. 

#### 3.3.3. Peri-Aortic Adipose Tissue

Analyzing the association between the cardiovascular risk factor and the characteristics of peri-aortic adipose tissue, we noted that patients with DM had a higher area and lower density of peri-aortic adipose tissue than patients without DM (area—Median = 257.0 mm^2^, IQR = 147.5, Median = 205.5 mm^2^, IQR = 164.0, *p* = 0.03; density—Median = −62.0 HU, IQR = 28.5, Median = −49.5, IQR = 35.8, *p* = 0.04). The density of peri-aortic adipose tissue was weak and negatively correlated with HbA1c (ρ = −0.22; *p* = 0.02). No correlation was found between smoking load and density or area of peri-aortic adipose tissue.

The multiple linear regression was also used to analyze the influence of DM, sex, age, statins and antiplatelet therapy in the area of peri-aortic adipose tissue. The results reveal that sex was a significant predictor of peri-aortic adipose tissue area (β = 0.26, t = 2.91, *p* = 0.00). The other variables had no impact. 

As described above, patients with DM had a higher area and lower density of peri-aortic adipose tissue, even after adjusting for sex [F(1,120) = 5.79, *p* = 0.02; F(1,119) = 5.14, *p* = 0.03, respectively].

### 3.4. Adipose Tissue and Anthropometric Measurements

#### 3.4.1. Subcutaneous Adipose Tissue

In this studied population, positive and moderate correlations were found for subcutaneous adipose tissue area with BMI and WC (BMI: ρ = 0.63, *p* = 0.00; WC: ρ = 0.63, *p* = 0.00). A positive and strong correlation was found between subcutaneous adipose tissue area and hip circumference (hip circumference: ρ = 0.70, *p* = 0.00; WHR: ρ = 0.13, *p* = 0.14; 4-sites skinfold thickness: ρ = 0.49; *p* = 0.00). The correlation between the subcutaneous adipose tissue density and anthropometric measures was weak or inexistent (BMI: ρ = −0.14, *p* = 0.11; WC: ρ = −0.25, *p* = 0.00; hip circumference: ρ = −0.21, *p* = 0.02; WHR: ρ = −0.13, *p* = 0.14; 4-sites skinfold thickness: ρ = −0.02, *p* = 0.82).

#### 3.4.2. Visceral Adipose Tissue

Positive and moderate correlations were found for visceral adipose tissue area with BMI and hip circumference (BMI: ρ = 0.59, *p* = 0.00; hip circumference: ρ = 0.42, *p* = 0.00). A positive and strong correlation was found between visceral adipose tissue area and WC (WC: ρ = 0.72, *p* = 0.00; WHR: ρ = 0.56, *p* = 0.14; 4-sites skinfold thickness: ρ = 0.22, *p* = 0.00). The density of visceral adipose tissue was negatively and moderately correlated with WC (BMI: ρ = −0.35, *p* = 0.00; WC: ρ = −0.44, *p* = 0.00; hip circumference: ρ = −0.26, *p* = 0.00; WHR: ρ = −0.35, *p* = 0.00; 4-sites skinfold thickness: ρ = −0.10, *p* = 0.27).

#### 3.4.3. Peri-Aortic Adipose Tissue

The area and the density of peri-aortic adipose tissue were weakly correlated with BMI and WC (area—BMI: ρ = 0.33, *p* = 0.00; WC: ρ = 0.35, *p* = 0.00; hip circumference: ρ = 0.25, *p* = 0.01; WHR: ρ = 0.18, *p* = 0.06; 4-sites skinfold thickness ρ = 0.17, *p* = 0.06; density—BMI: ρ = −0.33, *p* = 0.00; WC: ρ = −0.39, *p* = 0.00; hip circumference: ρ = −0.20, *p* = 0.03; WHR: ρ = −0.27, *p* = 0.01; 4-sites skinfold thickness: ρ = −0.11; *p* = 0.26).

### 3.5. Adipose Tissue and Medication

The area and the density of peri-aortic adipose tissue were weakly correlated with BMI and WC (area—BMI: ρ = 0.33, *p* = 0.00; WC: ρ = 0.35, *p* = 0.00; hip circumference: ρ = 0.25, *p* = 0.01; WHR: ρ = 0.18, *p* = 0.06; 4-sites skinfold thickness: ρ = 0.17, *p* = 0.06; density—BMI: ρ = −0.34, *p* = 0.00; WC: ρ = −0.39, *p* = 0.00; hip circumference: ρ = −0.20, *p* = 0.03; WHR: ρ = −0.27, *p* = 0.01; 4-sites skinfold thickness: ρ = −0.11, *p* = 0.26).

## 4. Discussion

In this research work, we found that the visceral, the subcutaneous and the peri-aortic adipose tissue compartments have distinct characteristics and associations with cardiovascular risk factors and anthropometric measures. Medication had different impacts in the distinct adipose tissue compartments. 

### 4.1. Characteristics of Visceral, Subcutaneous and Peri-Aortic Adipose Tissue Depots in CT Scan

We found that the area of the visceral adipose tissue was correlated with both the area of the peri-aortic adipose tissue and the area of subcutaneous adipose tissue, which is consistent with the literature. Fox et al. also reported a positive correlation between the subcutaneous adipose tissue area and the visceral adipose tissue area [31]. Lehman et al. described that the peri-aortic fat area in the thoracic aorta was correlated with visceral adipose tissue area, and Rapolti et al. found a correlation between the area of the peri-iliac adipose tissue and visceral adipose tissue area [32,33]. The correlation between the subcutaneous and the peri-aortic adipose tissue area was almost inexistent, as shown by Rapolti et al. [33].

Another important and less extensively studied piece of information regarding adipose tissue that can be obtained from the CT scan is the fat density, which is a measure of fat quality [34]. A less lipid-dense fat tissue is associated with a more adverse metabolic profile [34]. Some causes of lower adipose tissue density have been advocated for: an increase in adipocyte sizes (hypertrophy) [35], a decrease in the number of small adipocytes (hyperplasia) [35], less fibrosis [36], lack of blood vessels and vascularity [34]. The balance of these different elements dictates a higher or lower density. In visceral adipose tissue, an increase in area and a decrease in density result from larger hypertrophic adipocytes [37].

We registered that the subcutaneous adipose tissue had the lowest density, while the peri-aortic adipose tissue had the highest density (these differences were statistically significant). The density of the visceral adipose tissue is strongly correlated with the density of the peri-aortic adipose tissue and with the density of subcutaneous adipose tissue. The correlation between the subcutaneous and the peri-aortic adipose tissue density is almost inexistent. These data corroborate a strong relationship between the characteristics of the visceral tissue and the PVAT. The subcutaneous tissue and the peri-aortic adipose tissue compartments are quite different. The review of the literature did not find any study comparing density between visceral, subcutaneous and peri-aortic adipose tissue. However, it has already been demonstrated histologically that PVAT and visceral adipose tissue have a more pro-inflammatory state than the subcutaneous depot [38]. Measuring the levels of leptin and adiponectin in subcutaneous tissue and PVAT, Mauro et al. also suggested that these compartments have a distinct biologic role [39]. The highest density being found for peri-aortic adipose may be explained by the higher number of vessels (due to its proximity to the abdominal aorta) and by the higher inflammation in the PVAT. The lowest density being found for the subcutaneous adipose tissue is probably explained by the lower inflammation in this depot [39]. An increase in fat density is a marker of inflammation [40].

### 4.2. Characteristics of Adipose Tissue Depot and Cardiovascular Risk Factors

Analyzing the association between the amount (area) of adipose tissue and cardiovascular risk factors, we found that smokers/ex-smokers had a lower quantity of subcutaneous adipose tissue and patients with hypertension had a larger visceral adipose tissue area. These data are in accordance with the literature [41,42,43]. We reported a negative and moderate correlation between smoking load and subcutaneous adipose tissue area, which can be explained by lipogenesis inhibition in the subcutaneous region of the body caused by nicotine inhalation [44]. Smoking cessation causes an increase in body fat mainly in the subcutaneous region [44]. 

However, in this study, the association between smoking habits and subcutaneous adipose tissue seems to be explained by the confounder factor sex. It is well known that women have more subcutaneous adipose tissue [45]. In fact, this association was lost after adjusting for sex.

In our research work, patients with DM had more visceral adipose tissue, but this difference was not significant. The association between the quantity of visceral adipose tissue found by CT scan and DM has already been described [29,32]. We recorded that patients with hypertension had a higher visceral adipose tissue area than patients without hypertension, as reported by other authors [31].

A positive and weak correlation was found between serum triglycerides and visceral adipose tissue area, as found by other authors [31,45]. Sadeghi et al., in a study conducted on patients with coronary disease, also found an association between visceral adipose tissue area found by CT scan and other lipid molecules, such as total cholesterol and LDL cholesterol [46]. 

In our study, a positive correlation was found between visceral adipose tissue area and the number of cardiovascular risk factors, as shown in the general population studies [41]. The association between the number of cardiovascular risk factors and the subcutaneous area is very weak, as has been described in a few publications [34]. In line with our results, Fox et al. also concluded that visceral adipose tissue area was more strongly correlated with most metabolic risk factors than subcutaneous adipose tissue area [31].

Investigating the correlation between the cardiovascular risk factors and adipose tissue density, we found that smoker and ex-smoker patients had a subcutaneous adipose tissue of less density when compared to non-smokers. These data reinforce the negative role of smoking in the subcutaneous adipose tissue but not in the visceral compartment. The impairment of adipocyte hyperplasia caused by nicotine inhalation can be a physiopathological explanation for this fact [44]. We observed in the immunohistochemical analysis that smokers had a higher prevalence of CD 163+ macrophages in their subcutaneous adipose tissue. Consequently, we can hypothesize that smokers have subcutaneous fat with higher inflammatory cells and large adipocytes.

Serum triglycerides were negatively correlated with visceral adipose tissue density, as shown by Abraham et al. [34]. 

In our study, patients with DM had a higher subcutaneous adipose tissue density, which also was noted by Rosenquist et al. [36]. According to these authors, a higher fibrosis in subcutaneous adipose tissue of diabetic patients can be the explanation for these data [36].

The scientific community has paid attention to the role of PVAT. In our study, we evaluated the peri-aortic adipose tissue with CT scanning. Patients with DM had more but less dense peri-aortic adipose tissue, suggesting the presence of larger hypertrophic adipocytes. As far as we know, no study has reported that patients with DM have a higher quantity of peri-aortic adipose tissue around the abdominal aorta, but it has been described that diabetic subjects have more peri-aortic adipose tissue around the thoracic aorta [47]. Ömer et al. concluded that the quantity of adipose tissue that surrounds the thoracic aorta was positively correlated with HbA1c [48].

A negative and weak correlation was found between HbA1c and peri-aortic adipose tissue density, suggesting that worse glycemic control can be associated with a more adverse metabolic profile in PVAT and larger adipocytes. We did not find any study reporting the effect of DM or diabetic control on the peri-aortic adipose tissue quality.

Since the peri-aortic adipose tissue can influence the aorta and the atherosclerotic process, this result can have clinical implications, reinforcing the importance of a strict glycemic control in diabetic patients. Besides the traditional adipose tissue depots (visceral, subcutaneous, and PVAT), the adipose tissue is also stored in ectopic organs such as the liver, and this deposition is associated with cardiovascular risk, as proved by Cicero et al. Liver steatosis can give an accurate estimation of cardiovascular risk associated with arterial stiffness. This result is relevant, as this could lead to starting treatment in an earlier phase of cardiovascular disease [49].

### 4.3. Adipose Tissue and Anthropometric Measurements 

Although the image methods are the most reliable strategy to measure the quantity of adipose tissue, there is a correlation between anthropometric measures and the amount of fat quantified by CT scan. In our study, a positive and strong correlation was found between subcutaneous adipose tissue area and hip circumference. A positive and strong correlation was also registered between visceral adipose tissue area and WC. However, positive and moderate correlations were also found for subcutaneous adipose tissue area with BMI and WC. Positive and moderate correlations were found for visceral adipose tissue area with BMI and hip circumference. Consequently, WC and hip circumference are traditionally the best anthropometric measures to determine the visceral and the subcutaneous adipose tissue compartments, respectively. However, WC and hip circumference also estimate the amount of subcutaneous and visceral adipose tissue [31].

We recorded positive and moderate correlations of the anthropometric measures WC and BMI with peri-aortic adipose tissue area. This correlation has not been previously described in the PVAT of the abdominal aorta, but similar correlations have been reported in studies conducted on the adipose tissue surrounding the thoracic aorta [31,48,50]. 

We detected a negative and moderate correlation between the WC and the visceral adipose tissue density [36]. This correlation is consistent with other studies and can be explained by the adipocyte hypertrophy and the decrease in capillary density described in obesity [36]. Lower vascularization can lead to hypoxia and increased inflammation [34]. Patients with obesity may have a generalized inflammatory state.

### 4.4. Adipose Tissue and Medication

We discovered that patients taking statins had lower density of visceral adipose tissue, which can be explained by a reduction in inflammation in visceral adipose tissue that statins can cause, as previously highlighted by Kauerova et al. [51]. To the best of our knowledge, no study has previously reported the effect of statins on visceral adipose tissue density, although Raggi et al. showed that statins can reduce the epicardial adipose tissue attenuation [52].

Moreover, we noted that patients on ACEi/ARA had a lower visceral adipose tissue density, a finding that has not been reported in humans (as far as we know). A study on telmisartan in rats revealed that telmisartan has an anti-inflammatory effect on visceral adipose tissue, which could help to explain our results [52].

### 4.5. Clinical and Research Implications

In this work, we emphasized that peri-aortic adipose tissue has some common characteristics with visceral adipose tissue and is distinct from subcutaneous adipose tissue. This information can have clinical and research implications, as the treatments to optimize the characteristics of visceral adipose tissue could have an impact in peri-aortic adipose tissue. Peri-aortic adipose tissue, in particular, and PVAT, in general, seem to have a direct impact on the arterial wall, particularly in the atherosclerotic process. Tools that increase our understanding of PVAT can improve our knowledge about atherosclerosis. The visceral adipose tissue is more easily studied than peri-aortic adipose tissue. 

We also showed that CT scanning is a valuable tool to characterize the adipose tissue. CT scanning has been used to assess the size and distribution of the body’s fat stores, as well as to investigate the structure of the adipose tissue itself. Clinically, CT can be used to monitor changes in the size and distribution of adipose tissue over time. CT scanning also evaluates the density of adipose tissue, which is related to inflammation and adipocyte sizes and can be useful for obtaining clinical and research data. This information can have clinical implications in our daily practice, as many patients undergo a CT scan for diagnostic purposes, and it is possible to routinely and automatically evaluate adipose tissue characteristics using this exam. A systematic report on adipose tissue features could contribute to patient health education, as well as drawing attention to the importance of controlling the obesity epidemic among physicians. 

Additionally, this study emphasized the influence of medication on adipose tissue characteristics, potentially opening a new avenue for monitoring the effects of drugs on adipose tissue features such as area and density. The potential effect of statins and ACEi/ARA on the adipose tissue can influence clinical practice when choosing the medication for our patients.

### 4.6. Strength, Limitations and Future Perspectives

This paper provided a more comprehensive characterization of the distinct adipose tissue compartments and revealed some interesting connections between the adipose tissue and the cardiovascular risk factors. This scientific investigation has several strengths: it is a prospective work that included 177 “real life” patients and evaluated the adipose tissue in depth, yielding novel results. The number of patients included is sufficient to reach our objectives, as corroborated by our sample size analysis. We studied the morphologic characteristics of adipose tissue with CT scanning, using a rigorous method: the patients underwent a CT scan specifically parametrized for this purpose, and a semi-automatic technique was used in the adipose tissue quantification. We also analyzed the histologic features of the subcutaneous adipose tissue. We described the relationship between the densities of the subcutaneous, visceral and peri-aortic adipose tissue; we highlighted the impact of statins and ACEi/ARA on the density of visceral fat in humans; we analyzed the immunohistochemistry of subcutaneous adipose tissue samples and observed a higher number of inflammatory cells in smokers; and we described the impact of DM in peri-aortic adipose tissue. To our knowledge, some of these data have not yet been reported.

The main weakness of this work is that we studied a heterogenous white and old population with vascular diseases from a single center, and thus, the data may not be applicable to the general population. The majority of patients analyzed were male, and sex has an impact in body composition. The recruitment was not randomized or blinded and was based in clinical judgment. Numerous comparisons were made with few adjustments for possible confounding factors. We think that a multicenter and randomized study may overcome some of these constraints. 

Another limitation was the lack of a higher number of prior research studies focused on PVAT, as well as the absence of a consensual definition of PVAT limits in the CT scan. These facts limit the comparation between our results and those of other authors.

Additionally, we did not conduct a more comprehensive evaluation of the adipose tissue samples that we collected. We have samples of visceral tissue and PVAT in our biobank, and it is our plan to evaluate these samples and correlate them with the CT scan characteristics and with cardiovascular risk factors.

## 5. Conclusions

The peri-aortic adipose tissue is an adipose tissue compartment quite similar to the visceral adipose tissue and distinct from the subcutaneous adipose tissue depot. The cardiovascular risk factors have different influences in the distinct adipose compartments, with the subcutaneous fat density being directly affected by smoking habits. Medication such as statins and ACEi/ARA have a direct impact on the visceral adipose tissue density, suggesting that they can affect the inflammation in this depot.

## Figures and Tables

**Table 1:** Cardiovascular risk factors and comorbidities of the studied population.

	Studied Population (n = 177)
Male (n; %)	141; 80
Hypertension (n; %)	121; 68
Smoker/ex-smoker (n; %)	112; 63
Smoking load (PY)	28.15 ± 31.86
Dyslipidemia (n; %)	110; 62
Diabetes (n; %)	66; 37
HbA1c (%)	6.53 ± 3.01
Coronary artery disease (n; %)	23; 13
Statins (n; %)	143; 81
Fibrate (n; %)	14; 8
Ezetimibe (n; %)	9; 5
Antiplatelet (n; %)	126; 71
ACEi/ARA (n; %)	53; 30
Beta-blockers (n; %)	38; 21
Calcium channel blockers (n; %)	51; 29

PY: Pack-year; HbA1c: glycated hemoglobin; ACEi: angiotensin-converting-enzyme inhibitors; ARA: angiotensin II receptor antagonists.

**Table 2:** Cardiovascular risk factors and subcutaneous adipose tissue area (mm^2^).

	Present		Non-Present		*p*-Value
	Median	IQR	Median	IQR	
Hypertension	14,095.0	10,426.42	13,402.0	7419.8	0.33
Smoker/ex-smoker	13,330.5	6324.5	15,182.5	12,454.5	0.01 *
Dyslipidemia	14,273.5	8564.8	13,185.0	8917.0	0.27
Diabetes	15,030.0	9880.6	135,414.0	7273.5	0.28

IQR: interquartile range. * *p* < 0.05.

**Table 3:** Cardiovascular risk factors and subcutaneous adipose tissue density (HU).

	Present		Non-Present		*p*-Value
	Median	IQR	Median	IQR	
Hypertension	−103.6	880.0	−102.0	5.2	0.17
Smoker/ex-smoker	−104.0	5.0	−101.5	8.9	0.05 *
Dyslipidemia	−103.7	7.0	−102.0	5.1	0.15
Diabetes	−102.0	7.5	−104.0	5.0	0.00 *

IQR: interquartile range. * *p* < 0.05.

**Table 4:** Cardiovascular risk factors and visceral adipose tissue area (mm^2^).

	Present		Non-Present		*p*-Value
	Median	IQR	Median	IQR	
Hypertension	17,200.0	35,353.1	14,427.0	10,487.0	0.02 *
Smoker/ex-smoker	17,198.0	13,900.2	15,978.0	15,170.5	0.38
Dyslipidemia	15,978.5	12,656.0	17,528.0	14,312.0	0.74
Diabetes	17,532.0	13,191.0	15,396.0	13,774.5	0.10

IQR: interquartile range. * *p* < 0.05.

## Data Availability

Data will be provided by contacting the main author.

## Appendix A

1. Obtain a paraffin-embedded tissue sample from the desired area.
2. Section the paraffin-embedded tissue sample onto a slide at a thickness of 4–6 μm.
3. Turn on the bath.
4. Adjust the pH of the buffer, put it in plastic cuvettes and place them in the bath.
5. 3. Heat to 60 °C until the paraffin melts. (NOTE: if the slides are paraffinized, it requires more time).
6. Paraffin removal and hydration (Leica Auto Stainer XL): xylene, 2 × 5 min; absolute ethanol, 2 min; ethanol 95°, 2 min; ethanol 95°, 2 min; ethanol 70°, 2 min; water, 2 min.
7. Antigen recovery: bath 98 °C, 20 min (citrate buffer 10 mM); let cool for 20 min.
8. PBS wash, 2 × 5 min.
9. Inactivation of endogenous peroxidases: 3% in methanol, 10 min (stock H_2_O_2_ 30%).
10. PBS wash, 2 × 5 min.
11. Incubate with primary antibody 2 h at room temperature—CD45 abcam 10558, 1:500; CD163 Cell Marque™, MRQ-26 1:100 (manufactured by Cell Marque in California, United States).
12. Wash with PBS, 2 × 5 min.
13. Incubate with amplification solution (HiDef Detection™ HRP Polymer System, manufactured by Cell Marque in California, United States, and Cell Marque™), 10 min.
14. Wash with PBS, 2 × 5 min.
15. Incubate with polymer (HiDef Detection™ HRP Polymer System, Cell Marque™), 10 min.
16. Wash with PBS, 2 × 5 min.
17. Incubate with DAB (DAB Substrate Kit, produced in Massachusetts, United States of America, and Cell Marque™), 5 min.
18. Wash with H_2_O, 2 min.
19. Counter-stain with Hematoxylin (Leica Auto Stainer XL, manufactured by Leica biosystems in Nussloch, Germany).
20. Mount with Entellan in the Hotte.
